# Ninety-seven Years Old Hispanic Male With Mantle Cell Lymphoma

**DOI:** 10.4021/jocmr397w

**Published:** 2010-10-11

**Authors:** Beth Thompson, Maria Rosaida Gonzalez

**Affiliations:** a Department of Medical Education, Florida Hospital East Orlando Medical Plaza, 7975 Lake Underhill Road, Suite 200, Orlando, FL 32822, USA

## Abstract

Treating geriatric patients can be difficult. Patients often present with subtle or atypical symptoms, and end-of-life care is rarely neatly packaged. We present a case of a 97 years old Hispanic male with multiple medical problems who was diagnosed with mantle cell lymphoma after thoracentesis from what was thought to be a para-pneumonic effusion. An oncology evaluation determined that he was not a candidate for aggressive treatment. There is no evidence of curative treatment for mantle cell lymphoma. To complicate the decision making process further, the patient’s primary caregiver requested specifically that the health care team withholds the diagnosis from the patient. We discuss the inherent difficulties associated with end-of-life care.

**Keywords:**

Geriatrics; End-of-life care; Mantle cell lymphoma; Hispanic

## Introduction

Mantle cell lymphoma (MCL) is classified by the World Health Organization as a predominantly nodal mature B cell non-Hogdkins lymphoma (NHL). It can be an aggressive form of mature B cell lymphoma with a median survival of about 3 years [1, 2]. Risk factors for NHL include HIV, HTLV, EBV, H pylori infections, autoimmune diseases, congenital immunodeficiencies, and exposure to agricultural herbicides and pesticides or industrial solvents [3].

MCL often presents in late stages. Median age of diagnosis is 60 to 68. Patients typically (75%) initially present with lymphadenopathy, but can also present with extranodal disease. Common sites of involvement include the lymph nodes, spleen, Waldeyer's ring, bone marrow, blood, and extranodal sites, such as the gastrointestinal tract. One third of patients present with B symptoms of fever, night sweats and unintentional weight loss [2].

Our case highlights two important issues when treating geriatric patients with different cultural backgrounds. The first problem was the difficulty of diagnosis in an elderly male patient with multiple co-morbid conditions. The patient presented with shortness of breath and weakness which was suspected to be pneumonia with a para-pneumonic effusion. After thoracentesis, he was diagnosed with mantle cell lymphoma.

The second obstacle was communicating effectively with the patient and his daughter, the surrogate medical decision maker and primary caregiver. She informed the team that her father did not want to know of his diagnosis. This raised many ethical questions of how to treat the patient aggressively or refer for hospice effectively.

## Case Report

The patient was a 97 years old Hispanic male who was admitted to the hospital with shortness of breath and increasing fatigue. Two weeks prior to the current symptoms, the patient had been admitted, diagnosed with pneumonia, treated with antibiotics and then discharged home. Pertinent past medical history included hypertension, chronic obstruction pulmonary disease, anemia, recurrent clostridium difficile colitis, chronic kidney disease, osteoarthritis, cerebrovascular accident without deficit, hyperlipidemia, cholelithiasis, nephrolithiasis, abdominal aortic aneurysm, and colon polyps. Of note, the patient had been hospitalized 9 times in the past 12 months. Past surgical history included an appendectomy, repair of abdominal aortic aneurysm, and colonoscopy 2 years prior with unknown results. He had an allergy to penicillin. Family history was noncontributory. Current medications included moxifloxacin day 5, metroniadazole, amlodipine, omeprazole and losartan. Review of systems revealed weight loss, abdominal pain, and back, hip and leg pain.

Vital signs on admission were unremarkable. Remarkable examination findings included cachexia, scleral icterus, white coating on tongue, bibasilar rhonchi, rales and wheezes, diffuse bilateral lower quadrant pain, guiaic positive stool, and tenderness in the lumbar and gluteal regions. There was no evidence of lymphadenopathy.

Initial laboratory data revealed white blood cell count 12,200, hemoglobin 7.1, hematocrit 22.7, and platelets 291,000. Chemistry profile revealed BUN (blood urea nitrogen) 24, creatinine 1.34, glucose 134, and calcium 10.9. The remainder was unremarkable.

Chest radiograph on admission noted mild bilateral pleural effusions, left greater than right with bibasilar air disease not significantly changed from the week prior.

The patient was admitted with the following diagnoses: acute on chronic anemia secondary to a gastrointestinal bleed, pneumonia, chronic kidney disease, chronic obstructive pulmonary disease exacerbation, and oral candidiasis.

Over the next several days, as his multiple medical problems were addressed, the team noted a persistent left pleural effusion. A pulmonology consult was requested and thoracentesis performed. Pleural fluid analysis returned with features consistent with mantle cell lymphoma. FISH analysis was positive for a clonal BCL-1/IgH gene rearrangement. Flow cytometry revealed Kappa monoclonal B-lymphocytes co-expressing CD5 and CD19 with no CD23 expression. Cyclin D1 translocation was positive as well. At this point, hematology and palliative care consults were requested.

Computed tomography (CT) scan of chest, abdomen and pelvis revealed mediastinal adenopathy, diffuse gastric, pyloric and proximal duodenal wall thickening, as well as possible metastatic lesions in the pelvis with multiple ill-defined lucencies. Final diagnosis was metastatic Stage IV (Ann Arbor) Mantle cell lymphoma. With our patient’s advanced age, debilitated state and multiple medical problems, all members of the medical care team agreed with a hospice referral. The care team presented the recommendation to the patient’s caregiver.

The primary care and the palliative care team met with the patient’s daughter several times during the hospitalization to discuss her father’s diagnosis and prognosis. The team struggled to elucidate the role of hospice for her and, through her, for the family members not present. Family dynamics played a major role in the primary caregiver’s ambivalence toward hospice. Hospice was presented as a way for her father to stay at home and continue to be comfortable. She would have to help as necessary. The patient was discharged home to the care of his daughter and hospice. He is currently still alive and receiving regular physician home visits. He has not been told about the nature of his diagnosis. When the patient was asked by his PCP if he wanted to know the diagnosis, he continued to refer to his oldest daughter.

## Discussion

This case highlights two common problems in geriatric medicine. One is the difficulty in sorting through multiple medical problems to arrive at a relatively uncommon diagnosis, as the patient did not present with classic lymphadenopathy or have all the B symptoms.

The other challenge was interacting with the patient and family to make the best quality of life decision for the patient. The most important risk factor for poor prognosis in MCL is age more than 60 years. However, age should not be the only reason to avoid aggressive treatment. Other prognostic indicators include elevated serum lactate dehydrogenase level, advanced stage disease, decreased functional performance, hemoglobin, gender, and more than one site of extranodal disease [3]. There are several studies indicating that older adults in otherwise good health can tolerate the effects of the treatment which includes chemotherapy, radioimmunotherapy, and hematopoietic stem cell transplantation. However, there is no evidence for a cure [4, 5].

There are inherent conflicts that arise during these discussions. Caregivers need time to see how the patient is progressing, time to discuss matters with other family members, and time to grieve over the prospect of losing a family member. Physicians and hospitals are motivated to make decisions rapidly and move the patient to the final disposition.

Attitudes toward end-of-life care differ between ethnic groups which can be complicated by differences in ethnicity of the physicians as well. While most patients in all ethnic groups in a medicare survey wished to die at home, a higher percentage of Hispanic patients vs non-Hispanic Whites wanted to die in the hospital, who would tolerate drugs that entailed daily suffering to prolong life and wanted mechanical ventilation for life extension [6].

Braun et al discussed the challenges that health care surrogates have at the end of life. All ethnic/racial groups reported a tremendous burden. This was worsened if the prognosis was unclear and if the patient’s wishes were not known. If the surrogate could refer to the patient’s previously stated wishes, this burden was eased. Surrogate health care decision makers report a tremendous amount of stress involved in making the perceived ‘right’ decision and the possibility of being judged by other family members later [7].

A review of the literature performed by Kwak et al revealed that Hispanics are more likely to have advance directives than African-Americans, but less likely than Caucasians or Asian-Americans. Although all groups were found to have misconceptions about advance directives, Caucasians typically were more aware of advance directives. Some ethnic groups, such as Chinese and Navajo, were opposed to the discussion of death because of the perceived negativity inherent in the topic. Regarding life support, Hispanics tend to be more likely than Caucasians to prefer life support. In this paper, Koreans and Mexican-Americans tended to not want to be told of their diagnosis [8].

Hispanics prefer family-centered decision making. They are less likely to name only one health care proxy because it may exclude other members of the family. They were found to expect the adult children to formulate the decisions, thus removing the burden from the patient. This was born out with our patient and his family. With the removal of the burden from the patient, it gets squarely placed with the caregiver. In Hispanic families, this is most likely a daughter. Asian families tended to rely on their oldest son and daughter-in-law. Caucasians are most likely to rely on a spouse and African-Americans a family member [8, 9].

### Conclusions

This case stresses common challenges of working with elderly patients of various cultural backgrounds. Malignancy must be considered in a patient who is elderly and debilitated, even in the presence of multiple co-morbidities. A more timely diagnosis may not have changed our patients prognosis, but it could have saved him and his daughter multiple hospitalizations.

It is important to be sensitive to the needs of the family, especially the surrogate decision makers. Some prior knowledge of the cultural specifics can be helpful. Ideally, end-of-life decisions would be discussed prior to a major life shortening illness. The families of patients need time: to process their feelings of grief and loss, to understand a confusing or changing diagnosis, to communicate with other family members who may or may not be supportive of their decisions. It is important for the health care team to appreciate the fluidity of the familys response. This requires patience and practice speaking in clear terms. Taking these steps may make the experience more satisfying for both physicians and patients as well as patients families.

## Abbreviations

NHL: Non-Hodgkins Lymphoma
CT: Computed Tomography
MCL: Mantle Cell Lymphoma
HIV: Human Immunodeficiency Virus
HTLV: Human T-lymphotrophic Virus
EBV: Epstein-Barr Virus
H pylori: Helicobacter pylori
FISH: Fluorescence in situ hybridization
